# WSN-Based Multi-Sensor System for Structural Health Monitoring

**DOI:** 10.3390/s25144407

**Published:** 2025-07-15

**Authors:** Fatih Dagsever, Zahra Sharif Khodaei, M. H. Ferri Aliabadi

**Affiliations:** Department of Aeronautics, Imperial College London, South Kensington Campus, City and Guilds Building, Exhibition Road, London SW7 2AZ, UK; z.sharif-khodaei@imperial.ac.uk (Z.S.K.); m.h.aliabadi@imperial.ac.uk (M.H.F.A.)

**Keywords:** structural health monitoring, multi-sensor system, Bluetooth LE, WSN-based monitoring

## Abstract

Structural Health Monitoring (SHM) is an essential technique for continuously assessing structural conditions using integrated sensor systems during operation. SHM technologies have evolved to address the increasing demand for efficient maintenance strategies in advanced engineering fields, such as civil infrastructure, aerospace, and transportation. However, developing a miniaturized, cost-effective, and multi-sensor solution based on Wireless Sensor Networks (WSNs) remains a significant challenge, particularly for SHM applications in weight-sensitive aerospace structures. To address this, the present study introduces a novel WSN-based Multi-Sensor System (MSS) that integrates multiple sensing capabilities onto a 3 × 3 cm flexible Printed Circuit Board (PCB). The proposed system combines a Piezoelectric Transducer (PZT) for impact detection; a strain gauge for mechanical deformation monitoring; an accelerometer for capturing dynamic responses; and an environmental sensor measuring temperature, pressure, and humidity. This high level of functional integration, combined with real-time Data Acquisition (DAQ) and precise time synchronization via Bluetooth Low Energy (LE), distinguishes the proposed MSS from conventional SHM systems, which are typically constrained by bulky hardware, single sensing modalities, or dependence on wired communication. Experimental evaluations on composite panels and aluminum specimens demonstrate reliable high-fidelity recording of PZT signals, strain variations, and acceleration responses, matching the performance of commercial instruments. The proposed system offers a low-power, lightweight, and scalable platform, demonstrating strong potential for on-board SHM in aircraft applications.

## 1. Introduction

Structural Health Monitoring (SHM) refers to the continuous observation and evaluation of the condition of a structure using integrated sensor systems during operation. SHM addresses the growing need for efficient maintenance strategies in modern engineering fields such as aerospace, civil infrastructure, and transportation. The primary goals of SHM are to improve the safety of structures, minimize the maintenance-related inoperative period, and reduce the unnecessary mass associated with over-designed components [1,2]. These objectives are increasingly critical given the economic and environmental pressures faced by industries to optimize resource use while maintaining high-reliability standards. Lightweight structures, particularly in aerospace and transportation, require precise monitoring due to uncertainties in loading conditions, environmental influences, and material properties, which can lead to design redundancy and costly inspections [3]. SHM provides a pathway to predictive maintenance strategies, reducing safety-related inspections and allowing condition-based repairs that ensure structural reliability and regulatory standards [4].

Given these increasing demands on SHM systems, there is a growing need for technologies that offer improved scalability, reduced installation complexity, and real-time monitoring capabilities. Hence, the integration of Internet of Things (IoT) into SHM represents a promising approach for addressing some of the long-standing limitations associated with traditional SHM methods. Conventional wired SHM systems, while reliable, require extensive infrastructure, fixed installations, and cause high installation and maintenance costs, limiting their applicability to critical structures and long-term monitoring scenarios [5,6]. Wireless communication technologies, and specifically WSNs, emerged as an alternative to reduce these costs, enabling sensor nodes to transmit data wirelessly within infrastructure-less environments [7]. IoT-based SHM systems utilize advanced wireless communication protocols, edge computing, and cloud storage to collect, transmit, and process diverse structural parameters such as strain, vibration, displacement, temperature, and humidity from distributed sensing nodes [8]. This connectivity allows real-time damage detection and decision making, supporting predictive maintenance strategies and improving infrastructure safety [8,9]. In addition, the downsizing of sensor nodes, low-power operation, and advancements in data analysis further enhance the suitability of IoT for SHM applications [10]. Therefore, the adoption of IoT in SHM is considered as a promising and scalable solution to extend the service life of monitored structures while minimizing maintenance costs and operational risks [8,9,10].

Although SHM has significant potential to improve structural reliability and maintenance strategies, there is no unique SHM solution that can detect all types of damage in metals and composites, they often have to be tailored to the type and severity of potential damage in a structure (e.g., delamination in composites or cracks in metals) [11,12]. For example, dynamic methods such as guided ultrasonic waves are sensitive to changes in stiffness but can be heavily influenced by environmental factors such as temperature and humidity, which introduce noise and reduce accuracy [13]. Static methods, such as strain-based monitoring using fiber optic sensors, are less susceptible to environmental variations but lack the ability to detect global structural changes [14].

Among these challenges, the environmental sensitivity of many SHM techniques remains a critical limitation to their effectiveness. For example, guided waves and electromechanical impedance methods are strongly affected by temperature changes, which change material properties and signal propagation, leading to false alarms or reduced detection capabilities [12,13,15]. Noise from operational environments, such as mechanical vibrations or electromagnetic interference, further complicates signal interpretation and often requires extensive signal processing to extract meaningful data [16].

To overcome these limitations, multi-sensor systems have emerged as a transformative approach in the field of SHM. Unlike conventional single-method systems, multi-sensor systems combine multiple SHM techniques, implementing various physical principles to provide complementary data that improve the accuracy and reliability of damage detection and evaluation [10,14]. By integrating these techniques, multi-sensor systems enable the simultaneous evaluation of global and localized structural changes, leading to more detailed damage assessments [17,18].

The integration of multiple sensor types not only enhances impact detection but also enables usage monitoring through load reconstruction and shape sensing while accounting for environmental influences. By combining data from PZT, strain gauge, and accelerometer sensors, it is possible to estimate applied loads and track structural deformations, facilitating a more comprehensive evaluation of composite structures [17]. Furthermore, the use of additional environmental sensor for the monitoring of temperature and humidity allows the system to compensate for external influences, improving the robustness of the analysis [13,19].

More information on multi-sensor SHM systems can be found in [11]. This review paper highlights the potential of multi-sensor SHM systems for comprehensive damage assessment in metal and composite structures. By integrating static methods such as strain sensing with dynamic methods and guided waves, these systems effectively detect, localize, and quantify damage under varying environmental conditions. Data fusion at raw, feature, and decision levels ensures robust evaluation of diverse sensor inputs, overcoming the limitations of single-method SHM. The importance and need for data fusion for multi-sensor systems are covered in these studies [20,21,22].

In recent years, various multi-sensor systems for SHM have been proposed, each offering unique features suited for different structural and environmental conditions. Some systems prioritize inertial measurements and tilt detection to monitor large civil structures, incorporating MEMS-based accelerometers and inclinometers with embedded controllers for real-time data processing and management [23]. Others combine multiple sensing modalities, such as guided wave-based inspections, optical fiber sensing, and piezoelectric transducers, to achieve broader diagnostic capabilities in a single platform [24]. Advanced methodologies have emerged that minimize the requirement of baseline references by integrating symbolic data analysis and clustering algorithms, improving the adaptability and real-time detection capabilities of SHM frameworks [25]. Energy efficiency and scalability are also key considerations, with some systems employing modular architectures, low-power components, and frequency-hopping spread spectrum communication to ensure reliable, long-term DAQ even in complex environments [26]. Multi-parameter sensory inputs, such as fiber optic strain sensors, chloride sensors, and resistivity probes, have expanded the scope of SHM to include material degradation and corrosive processes [27], and the use of compressed sensing techniques has enabled the reconstruction of high-fidelity signals from undersampled measurements [28]. Furthermore, emerging approaches have integrated semi-passive RFID-based sensors to reduce energy consumption while providing dynamic strain and acceleration measurements [29].

Despite these advances, current multi-sensor SHM systems generally face several limitations. Many solutions adopt a single-sensor modality or exclude important environmental parameters that are crucial for comprehensive diagnostics. For example, while a given system may be good at detecting inertial vibrations [23], it may lack environmental sensors needed to account for temperature or humidity effects, potentially reducing the accuracy of damage detection. Similarly, integrated solutions employing multiple sensing types often do not achieve a sufficiently compact form factor or may rely on the combination of multiple rigid PCBs, limiting their applicability in weight-sensitive platforms such as aircraft [23,24,26,29].

Power consumption and communication constraints further complicate large-scale deployments. Wireless communication capabilities may consume significant energy due to the old 5 V powered Bluetooth modules [23] and may not be suitable to establish extensive sensor networks that cover large structures due to their wireless communication topology [26,29]. These shortcomings in wireless communication result in limited scalability and continuous long-term monitoring.

Moreover, while some systems integrate multiple sensors, they may still fail to achieve true multi-sensor capability due to the deployment of the same type of sensor rather than offering different sensor types that provide more information about the structure and its operational environment [25].

The implementation of multi-sensor SHM systems involves several challenges, which are primarily due to the complexity of integrating diverse sensors, managing vast amounts of data, and ensuring system reliability. WSNs for SHM must address issues such as sensor deployment and decentralized data processing to maintain connectivity and accuracy under resource constraints [30]. Moreover, the synchronization of sensing data across distributed networks poses significant difficulties due to clock drift and hardware-induced delays, which require advanced synchronization algorithms [31]. Energy limitations also constrain the deployment of wireless sensors, which makes multi-source energy harvesting techniques vital for extending operational lifespan [32,33].

These collective shortcomings, such as large dimensions, heavier configurations, the lack of environmental sensors for compensation, elevated power consumption, and limited sensing diversity, underscore the need for a more integrated and refined solution. This work addresses these challenges by introducing a fully integrated, miniaturized, and WSN-based multi-sensor platform specifically designed for on-board SHM applications in the aerospace industry. The system features a flexible PCB (3 cm × 3 cm) that incorporates a PZT, strain gauge, accelerometer, and environmental sensor, offering comprehensive structural assessments while minimizing both size and weight. The incorporation of low-power Bluetooth LE communication further enables scalable wireless deployment, avoiding additional cabling and enhancing suitability for aircraft applications where weight and wiring complexity are critical concerns.

In addition to hardware developments, this work also presents a custom GUI for managing the entire DAQ and communication process. The GUI facilitates data collection from a central node within the WSN, providing capabilities for data visualization, analysis, and storage. This ensures a streamlined and user-friendly environment for monitoring and analyzing SHM data.

## 2. Principle of Multi-Sensor System for SHM

The proposed system is based on the idea of integrating several different sensing elements into a single compact platform. Constructed on a flexible PCB dimensioned at only 3 cm by 3 cm, the system combines a PZT, a strain gauge, an accelerometer, and an environmental sensor that is capable of measuring air temperature, humidity, and pressure. By integrating these sensors, it enables the observation of both mechanical and ambient factors that influence structural integrity and long-term performance.

Based on the proposed design, each sensing element plays a distinct role in monitoring critical structural and environmental parameters for SHM applications. The PZT element records the structural vibration that arises under loads and dynamic events. The strain gauge monitors variations in strain distribution, which can indicate potential fatigue or accumulation of damage over time. The accelerometer quantifies structural responses to external forces, such as aerodynamic effects or turbulence, thereby supporting the identification of shifts in dynamic characteristics. Complementing these measurements are the environmental parameters recorded by the combined sensor for temperature, humidity, and pressure, all of which influence the material properties and the behavior of the structure under various operating conditions.

To enable effective DAQ and analysis, the data from each sensor are collected by a compact on-board electronic unit, which processes the signals for subsequent wireless transmission. The inclusion of Bluetooth LE communication removes the need for extensive wiring, allowing for direct placement of the sensor node on key structural areas. Such a configuration can be reproduced across multiple locations to form a wireless sensing network, providing spatially distributed data critical for understanding and predicting the health of the aircraft over time.

This wireless capability is complemented by a design that prioritizes low power consumption and durability. Hence, this miniaturized system is designed to operate with low power requirements and withstand the demanding environment of flight operations. By achieving a balance between functionality, mass, and form factor, the platform supports continuous or periodic monitoring tasks.

**Figure 1:**
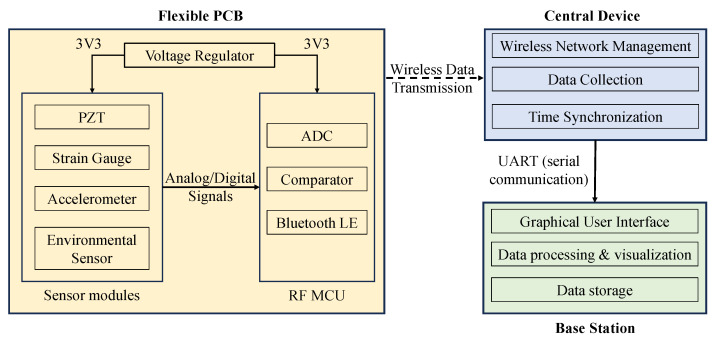
Conceptual block diagram of the miniaturized MSS for SHM.

In practice, these design principles allow multi-sensor systems to be strategically deployed on critical regions of composite structures, enabling comprehensive monitoring. All data acquired from the sensors available in the MSSs are transmitted through Bluetooth LE to the central device in the wireless network. This central device is connected to a base station, such as a PC. The base station is equipped with a GUI that can display these incoming sensor data in real time and save them for long-term study. The central device performs data processing steps to extract meaningful parameters from the raw signals. For example, it converts voltage measurements from the strain gauge circuit into strain values and extracts the peak voltage value from the PZT signal. The graphical interface then transforms these processed results into graphs and charts that allow one to examine variations in structural behavior over time. The design and operational principles of the proposed miniaturized MSS for SHM are represented in Figure 1.

## 3. Multi-Sensor SHM System

This section presents the architecture and key features of the multi-sensor SHM system, which is designed for wireless, low-power, and miniaturized structural health monitoring. It covers the RF MCU, which integrates Bluetooth LE for efficient data transmission, ensuring seamless communication across sensor nodes. The power management strategy is discussed, highlighting low-power operation, energy-efficient design, and battery selection for long-term functionality. The Sensors and DAQ subsection describes the integrated sensing elements, including PZT sensors, strain gauges, an accelerometer, and an environmental sensor, along with their roles in structural monitoring. Additionally, the Wireless Communication subsection outlines the antenna design and network topology, ensuring reliable data transmission for SHM applications.

### 3.1. RF MCU

The MSS incorporates Bluetooth LE communication, eliminating the need for external wireless modules. This integrated Bluetooth LE functionality allows for wireless data transmission between sensor nodes and a central data collection unit, enabling real-time monitoring without physical connections. Bluetooth LE communication supports a low-power, energy-efficient protocol, making it suitable for long-term operation in SHM applications.

The core of the system is the ANNA-B402 module from uBlox, which is built on the Nordic Semiconductor nRF52833 chip. This module integrates a high-performance Arm Cortex-M4 processor, providing efficient processing for signal acquisition, filtering, and real-time data analysis [34]. The ANNA-B402 module is a standalone Bluetooth 5.1 Low Energy solution, offering an ultra-compact form factor (6.5 × 6.5 × 1.2 mm) that makes it ideal for miniaturized systems.

By incorporating both processing and wireless communication within a single compact module, the MSS achieves an optimized balance between performance, power efficiency, and miniaturization.

### 3.2. Power Management

To ensure reliable and efficient operation, the MSS employs a DC-DC buck–boost voltage regulator, which provides a stable power supply when powered by either a coin cell battery or a lithium-ion/polymer battery. This regulator ensures a consistent voltage level, allowing the system to function efficiently under varying power conditions.

The system is designed with low-power operation strategies to extend the battery life. These strategies include the following:

- **Sleep Mode Implementation**: The MSS enters a low-power state during periods without impact detection, effectively reducing overall power consumption.
- **Threshold-Based System Activation:** When an impact-induced signal exceeds a predefined threshold value, the entire system is activated by the built-in comparator within the RF MCU. The ADC begins sampling PZT and strain gauge sensor signals, while the accelerometer measures acceleration, and the environmental sensor records air temperature, pressure, and humidity.

The choice of a CR-2032 coin cell battery is particularly beneficial for miniaturized SHM applications. This battery offers a capacity of 240 mAh at 3 V, which is sufficient to support the DAQ and wireless communication tasks in the proposed system. In addition, its small size (20 × 3.2 mm) makes it an ideal power source for compact embedded systems. While the CR-2032 is non-rechargeable, it presents a cost-effective alternative to lithium-ion batteries, which are typically larger, heavier, and more expensive.

By integrating power-efficient hardware components and software-driven energy optimization techniques, the MSS achieves long-term operation without frequent maintenance, making it well suited for SHM applications.

### 3.3. Sensors and DAQ

#### 3.3.1. Strain Gauge Sensor

The MSS incorporates a strain measurement module based on a quarter-Wheatstone bridge configuration, which is designed to detect small mechanical deformations in composite structures. The strain gauge sensor (120 ohm) is connected as one of the four resistive elements in the bridge, while the remaining three are fixed precision resistors (120 ohm).

To accurately measure the differential voltage produced by strain-induced resistance changes, the system employs an instrumentation amplifier (INA333 from Texas Instruments).

A precision voltage reference (REF35160 from Texas Instruments) supplies a stable low-noise reference voltage to both the Wheatstone bridge and the instrumentation amplifier, ensuring that any variations in the output are primarily due to mechanical strain rather than fluctuations in power supply.

**Figure 2:**
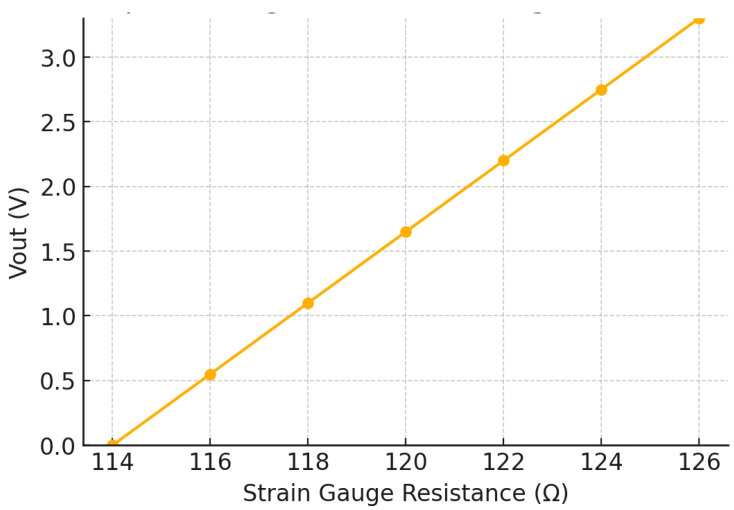
Output voltage vs. strain gauge resistance.

Once amplified, the strain signal is digitized using the ADC of the MCU, allowing for real-time monitoring and further processing. By combining a precision reference source, a stable bridge configuration, and a low-noise amplifier, the MSS achieves high-accuracy strain measurement, making it well suited for SHM applications. Based on the circuit configuration, the expected output voltage is presented corresponding to the resistor value of the strain gauge sensor, as shown in Figure 2.

#### 3.3.2. PZT Sensor

The PZT sensor module is designed to detect impact-induced signals and convert them into measurable voltage. To achieve this, the system employs a charge amplifier circuit based on an operational amplifier (LTC6087 from Analog Devices).

The raw PZT signal undergoes several conditioning stages:

- **Attenuation:** The signal is first attenuated by a factor of 2 with a voltage divider circuit to prevent saturation and maintain linearity.
- **Filtering:** A low-pass filter is applied with a resistor (51 ohm) and a capacitor (4.7 nF), resulting in a cutoff frequency of 664 kHz to remove high-frequency noise and improve signal clarity. If PZT signals are mostly less than 100 kHz, a cutoff around 500–700 kHz is acceptable. For filter circuit design and capacitor–resistor values, the reference design provided by Analog Devices was considered [35].

**Figure 3:**
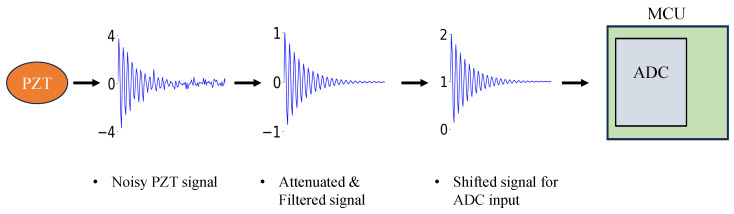
PZT signal acquisition stages.

After these adjustments, the processed analog signal is converted to digital form via the ADC of the MCU, allowing for real-time impact detection and further analysis. This ensures reliable impact sensing and improves SHM accuracy. The acquisition stages of the PZT signal are visualized in Figure 3.

#### 3.3.3. Accelerometer

The MSS integrates a BMA400 accelerometer from Bosch Sensortec, which is designed for low-power motion sensing applications. The accelerometer provides digital output via the Serial Peripheral Interface (SPI) protocol, ensuring efficient communication with the MCU. Using the SPI interface, the system benefits from fast and reliable data transfer, reducing latency in impact detection and motion analysis.

The BMA400 accelerometer supports the following [36]:

- Three-axis acceleration measurement, enabling comprehensive motion sensing and structural vibration analysis;
- Configurable measurement ranges of ±2 g, ±4 g, ±8 g, and ±16 g, allowing adaptability for different SHM applications;
- A 12-bit digital resolution, ensuring precise acceleration measurements essential for impact and vibration analysis;
- Adjustable output data rates of up to 800 Hz, optimizing the balance between power consumption and response time.

By integrating acceleration data with PZT and strain gauge measurements, the MSS provides a comprehensive evaluation of structural integrity, enabling real-time SHM applications. The accelerometer communicates with the MCU via the SPI interface, ensuring fast and efficient data transfer. The accelerometer provides real-time acceleration data, which are used for impact analysis, vibration monitoring, and structural response assessment.

#### 3.3.4. Environmental Sensor

The MSS incorporates a BME280 environmental sensor from Bosch Sensortec, which enables measurement of air temperature, atmospheric pressure, and humidity. Unlike PZT and strain gauge sensors, which provide analog outputs, the BME280 sensor features a fully digital output, eliminating the need for additional signal conditioning.

This sensor also communicates with the MCU via the SPI interface, ensuring fast and efficient data transfer. It supports the following features [37]:

- **Three environmental parameters:** temperature, pressure, and humidity;
- **High-resolution digital output:** 20-bit resolution for temperature and pressure and 16-bit resolution for humidity;
- **Power consumption:** Low-power operation, making it ideal for long-term monitoring applications.

By integrating the BME280 sensor, the MSS enhances its capability to correlate structural responses with environmental conditions, providing valuable insights for SHM applications. The collection of environmental data ensures that variations in temperature, pressure, and humidity can be accounted for in structural assessments, improving the accuracy and reliability of impact detection and damage evaluation.

**Figure 4:**
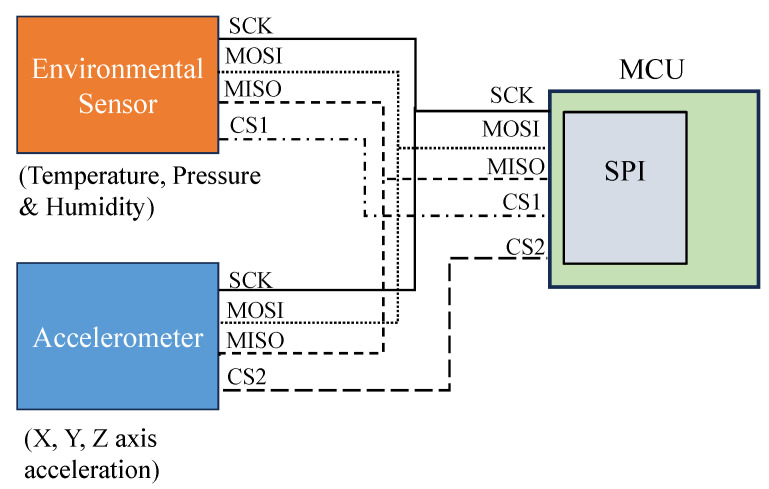
Serial communication (SPI) between MCU and sensors with digital output (BMA400 and BME280).

The MCU configures these sensors and reads these measurements through the SPI, which is a serial communication protocol. This protocol establishes a specific set of signals, including clock, input, output, and chip select lines, to enable efficient data transfer between the MCU and individual sensors, as shown in Figure 4. This approach eliminates the need for additional analog conditioning for these inputs.

### 3.4. Wireless Communication in MSS

The MSS utilizes an integrated antenna within the ANNA-B402 module, providing Bluetooth LE wireless communication for real-time SHM. The wireless network follows a star topology, where multiple sensor nodes communicate with a central unit. This structure enables efficient data collection, minimizes interference, and optimizes energy usage while ensuring reliable performance for SHM applications.

To achieve stable and interference-free wireless transmission, a custom antenna pattern was designed based on the integration guidelines of the uBlox system [38]. One of the critical design considerations is the implementation of a non-conductive keep-out area around the antenna. This area is maintained on both the top and bottom layers of the PCB, preventing signal reflections from nearby conductive components. Without this isolation, metallic traces or ground planes could interfere with the radiation pattern, significantly degrading signal strength and increasing data loss.

The key design considerations for antenna performance optimization include the following:

- **Unobstructed transmission path:** Ensuring that the antenna is not covered by metallic components, which could cause unwanted signal attenuation;
- **PCB layout optimization:** Properly routing traces to minimize interference and electromagnetic coupling between the antenna and other circuit components;
- **Ground plane design:** Implementing an appropriate ground clearance region to enhance the antenna efficiency and signal propagation characteristics.

By implementing these antenna design principles, the MSS ensures robust wireless data transmission, enhancing its applicability for SHM applications where reliable and efficient communication is crucial.

## 4. MSS System Implementation

**Figure 5:**
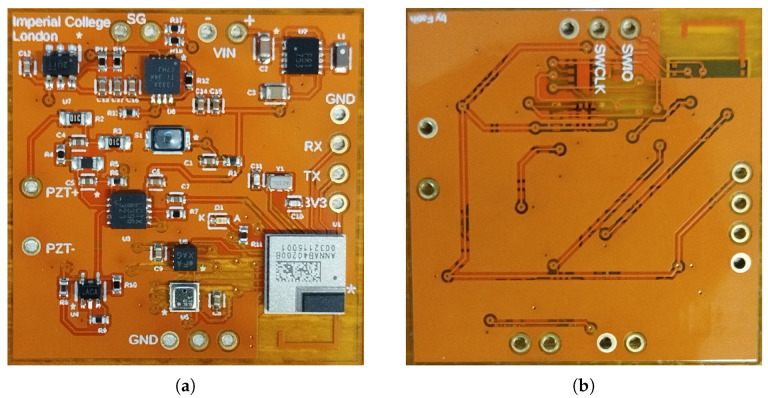
Multi-sensor SHM system in two-layer flexible PCB: (**a**) top view, (**b**) bottom view. The “*” symbol denotes the position of pin 1 on each component.

The proposed system was implemented on a flexible two-layer PCB to meet the miniaturization requirement, being a key design objective. Small-sized components were carefully selected to ensure they provided the necessary performance while minimizing the overall footprint. The dimension of the prototyped PCB is only 3 cm by 3 cm. The developed system is lightweight, weighing just around 0.5 g. Furthermore, the total cost of producing each MSS is approximately EUR 80, making it a cost-effective solution. The prototype of the developed system is presented in Figure 5. As shown in the figure, all components were placed on the top layer of the flexible PCB.

### 4.1. Function Modules in MSS

In the proposed MSS, There are six function modules to perform the sensing process for SHM. The first module in the system is the strain gauge module that converts mechanical strain into electrical signals. A quarter-Wheatstone bridge circuit was implemented with an instrumental amplifier to acquire the strain data from the structure. The second is the PZT module that includes the charge amplifier circuit that provides voltage outputs correlated with the vibrational energy. The PZT signal is completely shifted to the positive side using a reference voltage to be processed later by a microcontroller. The voltage output range of both the strain gauge and the PZT modules was adjusted from 0 to 3.3 V, allowing the ADC to properly sample and convert their electrical signals to digital values. Otherwise, a high-voltage input may permanently damage the ADC.

Unlike the strain gauge and PZT modules, both the accelerometer and environmental sensor modules have digital output for the sensors measurements. These sensor devices can be configured, and their measurements are read through the SPI serial interface. Using a single SPI interface, the MCU communicates with only one device at a time. To manage successful data reading from different devices, the chip selection pins are used to select the sensor required to communicate. While the environmental sensor measures air temperature, pressure, and humidity to support continuous monitoring of environmental parameters that may influence structural performance, the accelerometer is responsible for measuring acceleration in three axes.

Another critical module is the RF microcontroller, including the necessary peripherals in this study such as the ADC and the comparator. The ADC is responsible for sampling the signals coming from the strain gauge and PZT modules, while the comparator is used to enable an event-triggered mechanism in the proposed system. This module also manages serial and wireless communication protocols. The module supports Bluetooth LE to transmit data wirelessly to a central device that establishes the wireless network. While the SPI interface is used to communicate with the accelerometer and environmental sensor devices, the UART is also implemented for data exchange between the module and any device supporting this serial interface.

The power management module consists of the buck–boost DC/DC converter for voltage regulation to maintain stable electrical conditions across all components. A fixed voltage input (3.3 V) is provided to the system.

**Figure 6:**
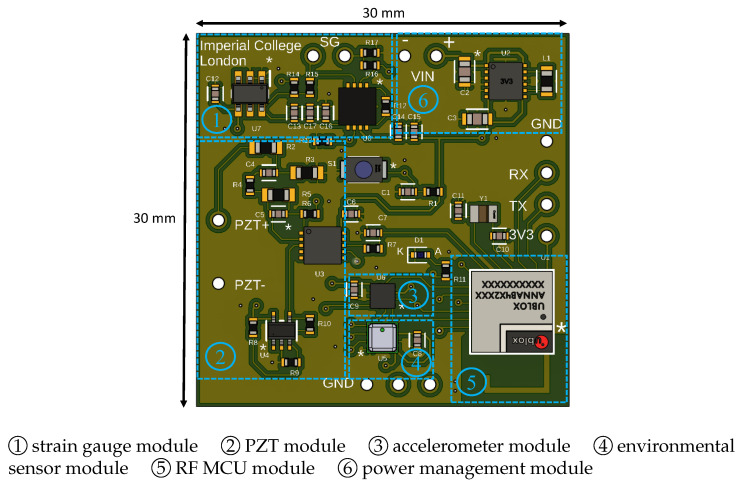
The function modules of multi-sensor SHM system. The “*” symbol denotes the position of pin 1 on each component.

All function modules mentioned above in the multi-sensor SHM system are represented in Figure 6.

### 4.2. Wireless Network Implementation in MSS

Bluetooth LE has been used to establish a star topology network characterized by its simplicity, compactness, and adaptability. This configuration is based on two primary device roles: central and peripheral. A central device functions as a hub, enabling connections to multiple peripheral devices and supporting data transfers up to the 2 Mbps limit, making it suitable for applications with high data demands [39]. To implement this topology, the Nordic UART Service (NUS) was used, which extends the central role of NUS to accommodate up to 20 peripherals. Further scalability was achieved through additional peripherals—termed the Multi-NUS solution. This extension was facilitated by software libraries such as the Connection Context Library, which simplified the configuration and evaluation processes.

Building on this framework, a WSN was developed using the star topology network and the Multi-NUS solution. To ensure secure wireless communication, the Filter Accept List method was used, restricting network access to devices explicitly included in the list. This approach ensures that only authorized and bonded devices can establish connections, enhancing the security and reliability of the network.

#### 4.2.1. Time Synchronization in MSS

In this study, time synchronization was also implemented in a wireless network. The proposed system ensures precise timing and synchronization in WSNs for SHM. A central node, acting as the timing master, transmits synchronization packets to peripheral nodes (timing slaves), which update their local timers to achieve a shared clock. Synchronization is implemented using Bluetooth LE on a single-RF channel (2480 MHz), with each node running a 16 MHz free-running timer. The timing master operates two timers—a continuously running synchronization timer and a counter to track overflows—while timing slaves adjust their timers upon receiving synchronization packets.

#### 4.2.2. Packet Structures for Sensor Data Handling

In the WSN developed for this study, an MSS serves as a fundamental node. Each MSS integrates multiple sensors to monitor various parameters, such as the PZT signal, strain, acceleration, environmental conditions, and timestamps. The network may contain several MSS nodes, all of which transmit data wirelessly to a central device. Effective communication within this network requires the ability to identify which MSS has transmitted a particular data packet and the type of data it contains. This identification is essential for ensuring accurate data categorization, processing, and interpretation, particularly in a system where multiple sensor nodes operate simultaneously.

To address this requirement, a structured data packet format was designed. Each packet begins with a byte identifying the MSS from which it originates. For example, a byte value of 1 in string format indicates that the data are from MSS 1. The second byte in string format specifies the type of data included in the packet. A value of 1 corresponds to ADC data, which can include PZT or strain gauge sensor readings; a value of 2 indicates timestamp data; 3 represents environmental sensor data such as temperature, pressure, and humidity; and 4 corresponds to accelerometer data. Each packet ends with a newline character (\n) to signify the conclusion of the data packet and enable proper processing by the central device.

The inclusion of a newline character is critical for ensuring that the central device can efficiently identify the end of each packet and separate incoming data streams. This differentiation enables the central device to verify that all data in the packet have been received before preparing the packet for transmission to a base station. While only the ADC data packet has a fixed length, others may have a different amount of data in the packet each time.

By combining a well-defined packet structure and unique identifiers for each data type, this system ensures that both the source and content of each packet are clearly identified within the WSN. This approach minimizes the risk of misinterpretation and improves the overall reliability and performance of the system.

Building upon the structured packet format described above, the specific format and content of each type of data packet are outlined below. Each packet type was customized to the specific sensor data it contained, ensuring efficient transmission and accurate interpretation by the central device.

**Figure 7:**
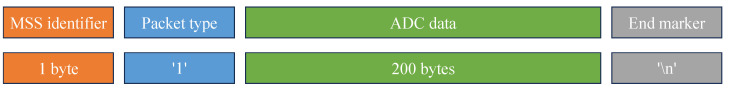
Format of ADC data packet.

**Figure 8:**
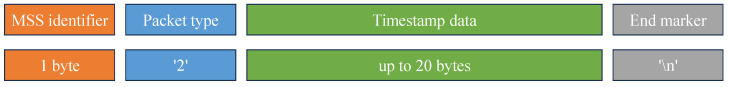
Format of timestamp data packet.

**Figure 9:**
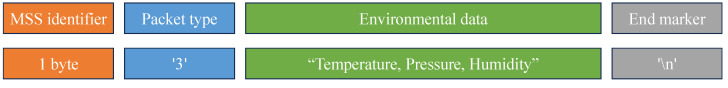
Format of environmental data packet.

**Figure 10:**
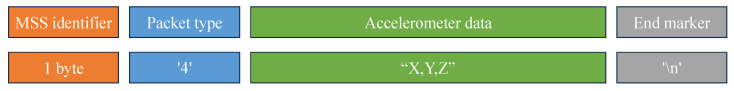
Format of accelerometer data packet.

- **ADC packet:** It begins with a byte identifying the MSS, followed by a byte specifying the packet type (value 1 for ADC data). The subsequent data block contains digital values that represent the readings of the PZT or strain gauge sensor. Each ADC packet contains sensor data of 200 bytes in integer format. The packet format is represented in Figure 7.
- **Timestamp packet:** It begins similarly, with a byte identifying the MSS and another byte indicating the data type (value 2 for timestamp data). The rest of the packet contains the timestamp of impact in microseconds and string format. The packet format is represented in Figure 8.
- **Environmental data packet:** After the MSS identifier byte followed by the byte indicating the packet type (value 3 for environmental data), the packet consists of temperature (°C), pressure (Pa), and humidity (%*RH*) respectively. The comma (,) is used as a delimiter to separate the different types of measurement in the packet. This allows the base station to easily distinguish and extract sensor data from the packet to properly process them. The packet format is represented in Figure 9.
- **Accelerometer packet:** In the same way, the packet begins with the identifier byte for the MSS, and then the second byte specifies the packet type (value 4 for accelerometer data). Following that, the X, Y, and Z axis data are respectively represented in integer format with 12-bit resolution and separated with commas in a similar manner to the environmental data packet. The packet format is represented in Figure 10.

## 5. Experimental Setups for Different SHM Applications

**Figure 11:**
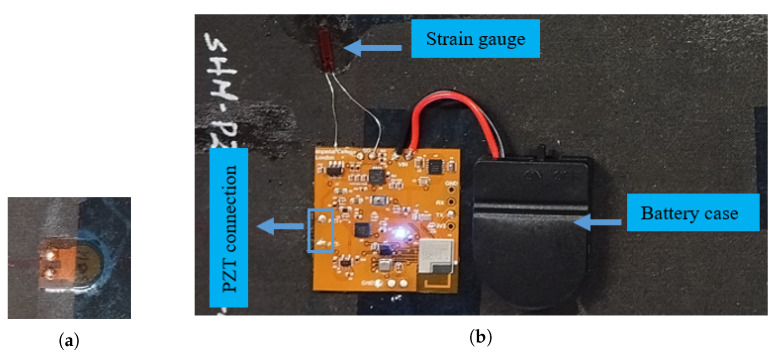
Multi-sensor SHM system on a composite panel: (**a**) PZT sensor, (**b**) MSS integration.

To integrate the multi-sensor SHM system to a structure, the PZT terminals available on the flexible PCB were soldered directly to a PZT sensor, as shown in Figure 11a, and the PZT sensor was placed under the flexible PCB to reduce the amount of wiring, as shown in Figure 11b. In addition to the PZT sensor, a strain gauge mounted on the structure was also soldered using the assigned terminals on the PCB. In contrast to other sensors, the accelerometer and environmental sensor were installed to be available directly on the PCB, eliminating the need for additional connections. Lastly, to power up the system, the power rails were soldered from the battery case, which housed the CR-2032 coin cell battery. The MSS we designed is completely functional for monitoring the health of the structure, as displayed in Figure 11.

### 5.1. First Experimental Setup

**Figure 12:**
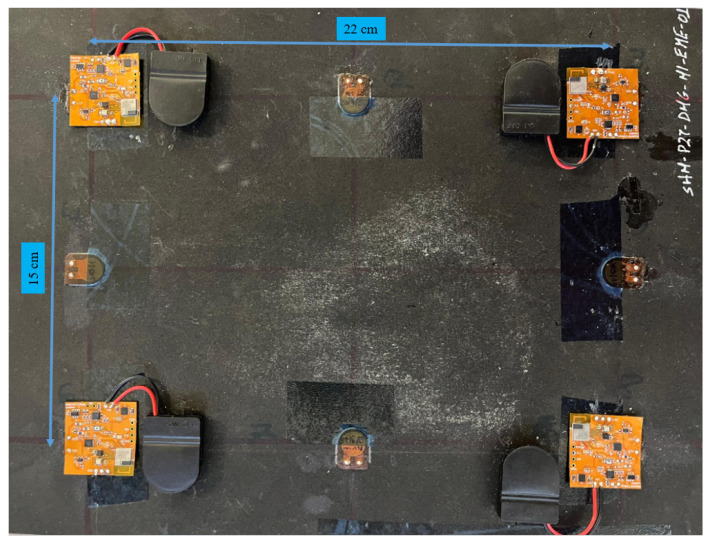
Multiple MSSs setup on a composite panel.

The first experimental setup consisted of a composite panel of dimensions 22 cm by 15 cm equipped with four multi-sensor SHM systems, as shown in Figure 12. In this example, the MSSs included strategically placed PZT sensors, an accelerometer, and an environmental sensor to capture comprehensive data for SHM applications. The MSSs acted as wireless sensor nodes in the WSN formed with Bluetooth LE. They were responsible for collecting and transmitting structural response data along with environmental information.

The setup aimed to evaluate the performance of the multi-sensor SHM system in detecting changes in the structural properties of the composite material under various conditions. Wireless communication was employed for remote monitoring and DAQ without physical connections, thereby reducing wiring complexity.

In the event of critical impact crossing predefined threshold values, each MSS is triggered and wakes up from the sleep mode immediately. The response of the structure is then recorded by the PZT sensor. Meanwhile, the timestamp of the impact is also captured from the shared clock in the wireless network. In addition, the acceleration in three axes is collected along with environmental condition measurements, which are temperature, pressure, and humidity.

There is a critical point where the comparator in the MCU is used to detect voltage transitions in the PZT sensor output. Its configuration includes a 64-level reference ladder to set the threshold voltage, and it generates interrupts in a high-speed mode to activate the system immediately. Because the PZT signal is shifted to the positive side, the system uses 1.65 V as the reference level around which the signal oscillates.

**Figure 13:**
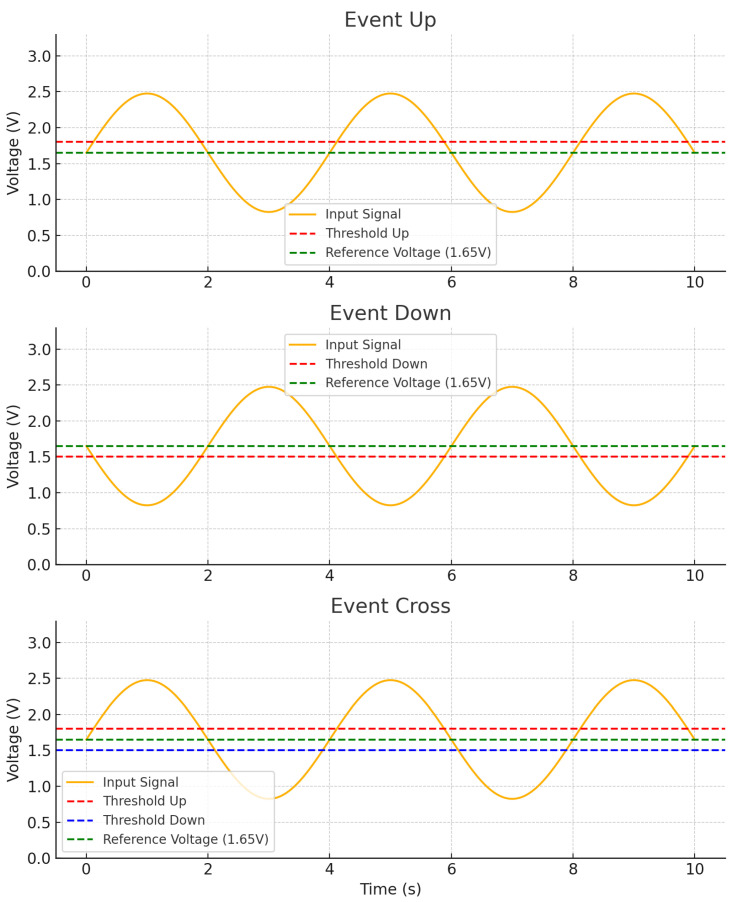
Comparator events with threshold aspect.

Based on the example plots shown in Figure 13, the comparator could be configured to generate either an Event Up or an Event Down, each triggered when the input signal crossed a single threshold level in the upward or downward direction, respectively. However, relying on only one threshold does not capture both possible directions of the PZT signal when it first crosses the reference. This may cause the system to be triggered with delay and miss some part of the initial signal. Instead, an Event Cross with two predefined threshold levels is needed. In this approach, both upward and downward trends of the PZT signal were monitored. As a result, the comparator output could accurately reflect the initial crossing event, regardless of whether the PZT signal moved above or below the reference at the beginning.

All data acquired after impact were wirelessly transmitted to the central device in the wireless network by each MSS. The central device sent all collected data packets to a base station, in this case a PC, through serial communication. These data packets were processed based on their packet types by the GUI running on the base station.

### 5.2. Second Experimental Setup

**Figure 14:**
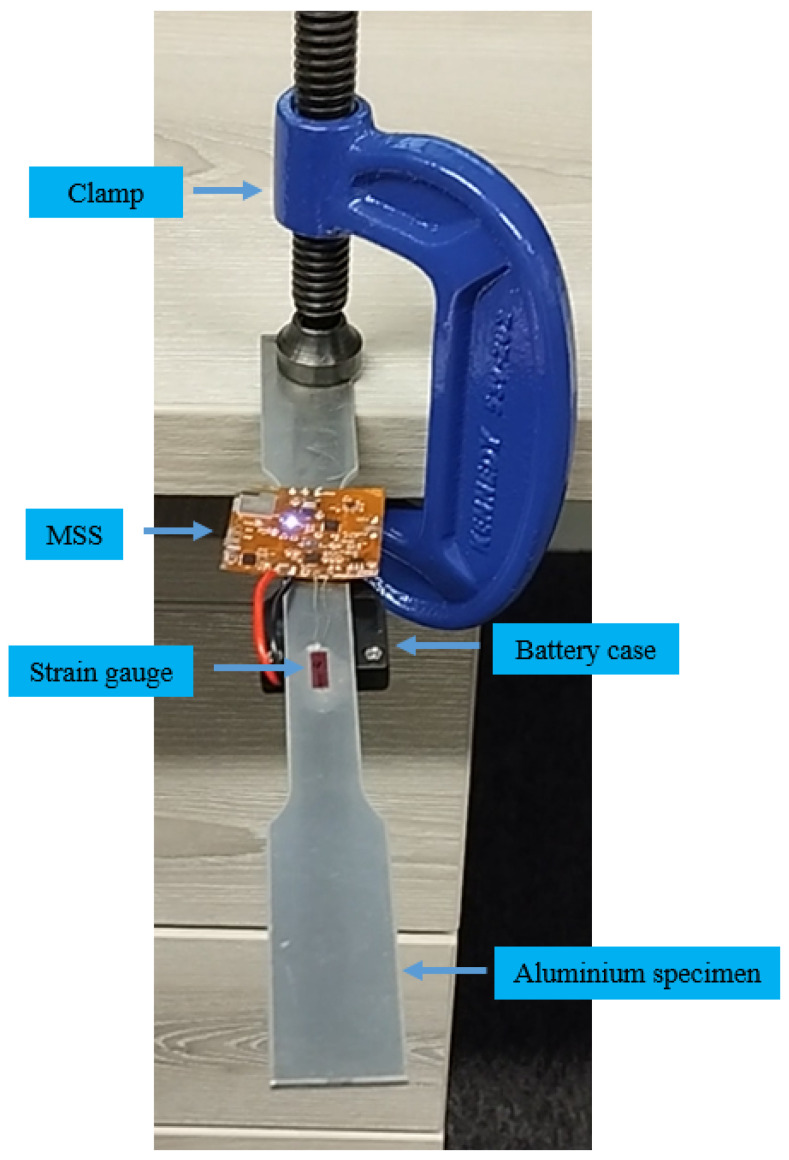
Experimental setup for strain measurement with aluminium specimen.

The multi-sensor SHM system was configured and programmed based on the requirements of an application, offering flexibility. In the second experiment, only the strain gauge and accelerometer sensors were used, and the strain and acceleration measurements were continuously recorded in the structure simultaneously. An aluminium specimen was used as shown in Figure 14 to be subjected to large deformation and observe a wider range of strain value, as composite panels are much stiffer. Therefore, the aluminium specimen was more suitable to assess the performance of the strain gauge and accelerometer modules in the MSS.

In this experiment, the strain gauge was placed in the middle of the aluminium specimen. While one side of the specimen was clamped on the desk, force was applied to another side upward and downward to observe changes in the strain. The accelerometer was integrated on the flexible PCB and was ready to record as well.

Before starting the experiment, there was a challenge that needed to be considered. That is, the offset in the strain gauge measurements is a common issue that requires calibration to ensure accurate and reliable data. This offset can result from various factors, including initial imbalances in the Wheatstone bridge due to resistor tolerances or inconsistencies in the bonding of the strain gauges to the structure. Temperature changes can also affect the resistance of the strain gauges and bridge components, leading to offset in the absence of any applied force. In addition, the signal conditioning circuit, particularly the amplifiers, may introduce inherent offset voltages. Residual stresses within the structure or fluctuations in the power supply to the Wheatstone bridge can further contribute to deviations in the baseline measurement. Environmental noise, such as electromagnetic interference, may also influence the observed offset.

To address these challenges, the calibration procedure in the system involved determining the offset when the structure was in a steady condition without any applied force. During this phase, 1000 initial samples were collected and averaged. The calculated average value was used to represent the offset. This offset was then subtracted from subsequent measurements to correct for any baseline error. This approach ensured that the strain gauge outputs were calibrated accurately, improving the overall precision of the measurements.

The GUI was modified for this experiment with new functionalities to calibrate the system and display real-time data streams coming from the serial port. The GUI provided a continuous visualization of the data, allowing its users to monitor the state of the system in real time.

## 6. Results and System Performance of MSS

The proposed multi-sensor SHM system is designed for versatile deployment. For example, it supports both event-triggered DAQ, where composite panels are impacted to acquire multi-modal sensor data, as well as continuous monitoring of real-time strain and acceleration. Its precise time synchronization further enables an accurate correlation of sensor outputs. The verification and validation of the system were carried out for different scenarios.

### 6.1. Application 1: Event-Triggered Experiment to Acquire All Types of Sensor Data

**Figure 15:**
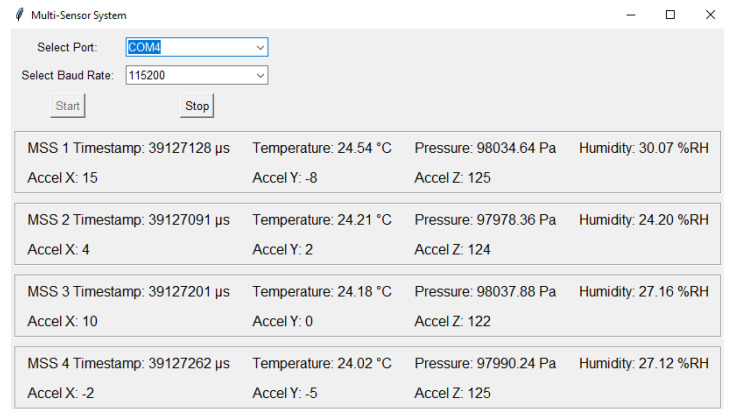
Timestamps and sensor data from four MSSs in the wireless network.

In the first experiment, impacts, which exceeded predefined threshold values, were made using a hammer on the composite panel, as shown in Figure 12. Afterwards, each MSS wirelessly transmited timestamp, PZT signal, accelerometer, and environmental sensor data to the central device. The GUI processed all packets coming from the central device, and each module was labeled (e.g., ‘MSS 1’, ‘MSS 2’, etc.). The concurrent outputs of multiple sensor modules were displayed, such as the timestamp in microseconds and the set of measurements of temperature (°C), pressure (Pa), humidity (%*RH*), and acceleration in the X, Y, and Z axes (12-bit digital format), as shown in Figure 15.

**Figure 16:**
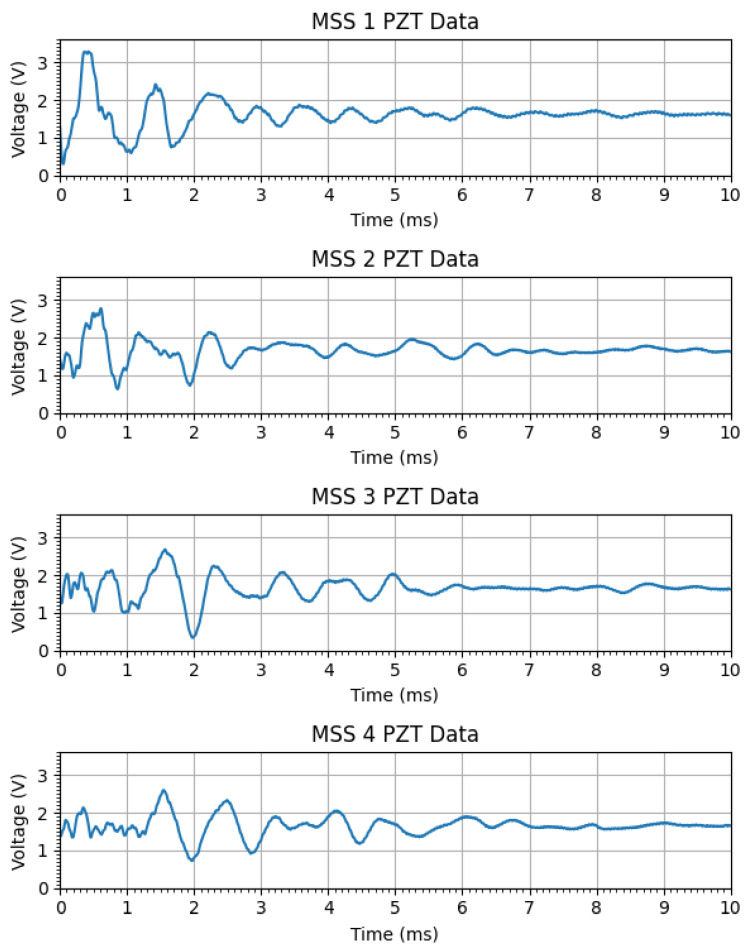
PZT signals from four MSSs in the wireless network.

In addition, the PZT signals generated by the impact were plotted and displayed in a different window, as shown in Figure 16. These PZT signals were plotted after a set of signal conditioning on the hardware side, such as filtering, attenuation, and shifting. Therefore, the signal voltage range was from 0 to 3.3 V and oscillated around 1.65 V, as configured in the circuit. For a data acquisition window of 10 ms in each MSS, 1000 samples were taken due to the sampling frequency of 100 kHz in the ADC.

### 6.2. Application 2: Real-Time Continuous Strain and Acceleration DAQ

**Figure 17:**
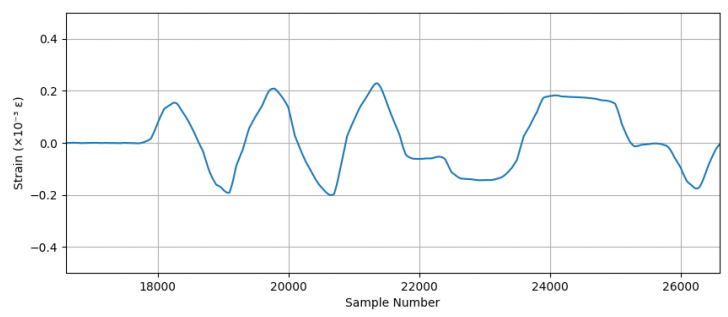
Real-time strain data display.

In the second experiment, only the real-time strain and acceleration measurements of the aluminium specimen were displayed and recorded. An example of the real-time data stream is shown in Figure 17 from the strain measurement.

When a force is applied to a specimen, the resulting strain depends on the direction of the force relative to the structure. The strain is a dimensionless measure of deformation, representing the relative change in length of a material under stress. It can be categorized into tensile strain (positive strain) and compressive strain (negative strain).

**Figure 18:**
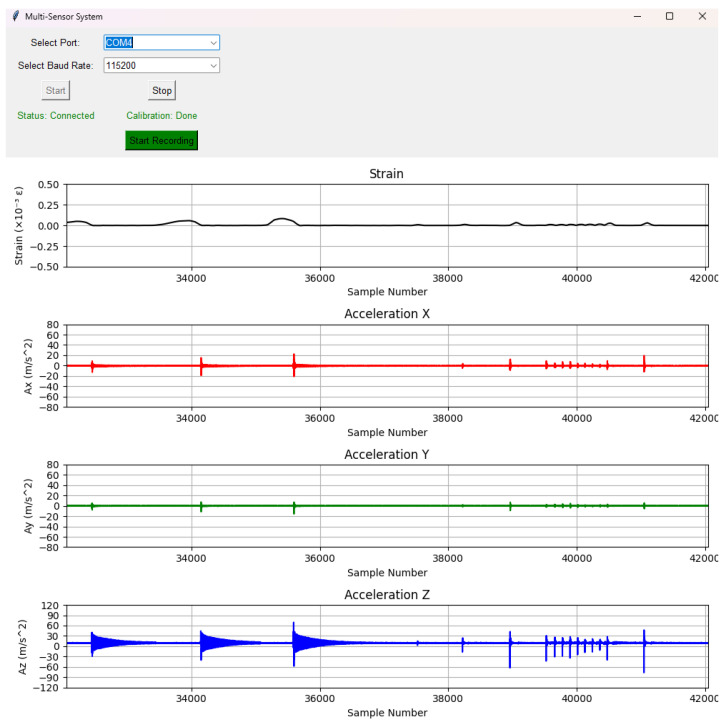
Real-time acceleration data display for three axes.

In addition, the accelerometer sensor measurement is also shown in Figure 18 separately for three axes. The results are expressed in meters per square second (m/s^2^).

Beyond its application capabilities, the performance of the proposed system was evaluated by comparing its measurement results against those obtained from commercially available instrumentation. Specifically, the acquisition of the PZT signal was evaluated by comparing it with data recorded using a digital oscilloscope configured with a matching resolution and sampling frequency. Similarly, real-time strain and acceleration measurements were validated using reference-grade devices. The strong agreement observed between the system output and those of the commercial instruments demonstrates its reliability in accurately capturing and transmitting sensor data.

### 6.3. PZT Signal Acquisition Performance

The precision of the proposed system in the acquisition of PZT signals was validated through a comparative evaluation with a commercial digital oscilloscope (TiePie Handyscope HS5). To ensure consistency across both platforms, the oscilloscope settings were configured to match the specifications of the MSS, specifically operating at a 12-bit resolution and a sampling frequency of 100 kHz.

Furthermore, the trigger thresholds of the oscilloscope were calibrated to reflect the MSS detection parameters. Given that the MSS signal conditioning circuitry introduces an attenuation factor of 2 and an offset of 1.65 V, the oscilloscope thresholds were set at −0.3 V and 0.3 V. These values correspond to MSS detection levels of 1.5 V and 1.8 V, thereby ensuring a consistent basis for comparison between the two systems.

For signal acquisition, the PZT sensor was connected in parallel to both the MSS and the oscilloscope, allowing for simultaneous recording of the same impact event under identical conditions. The excitation was introduced with a hammer strike, and both systems recorded the resulting waveform concurrently.

**Figure 19:**
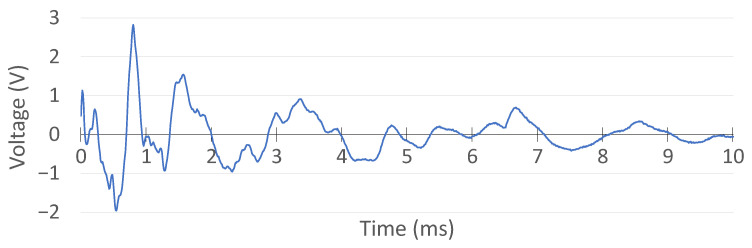
Digital oscilloscope measurement of the PZT signal.

**Figure 20:**
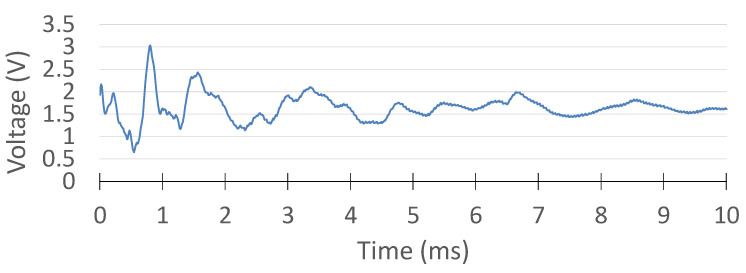
MSS PZT result.

The oscilloscope data were logged using its vendor-specific software interface, while the MSS data were transmitted wirelessly to a central unit and captured through a custom-developed GUI. The waveform acquired via the oscilloscope is presented in Figure 19, and the corresponding MSS signal is shown in Figure 20, enabling a direct visual comparison.

**Figure 21:**
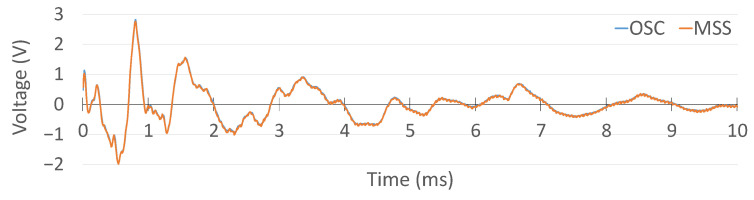
Comparison of the oscilloscope and MSS results.

Prior to comparison, the MSS output was post-processed to remove the 1.65 V offset and scaled to compensate for the factor-of-2 attenuation. The resulting aligned signals are displayed in Figure 21. The overlaid signals demonstrate an excellent match in both amplitude and temporal response, confirming that the MSS system is capable of acquiring and transmitting PZT signals with a fidelity equivalent to that of the commercial oscilloscope.

### 6.4. Continuous Strain and Acceleration Data Acquisition Performance

The capability of the proposed MSS for real-time strain and acceleration measurement was evaluated against a commercial DAQ system (Genesis HighSpeed DAQ hardware and Perception software from HBK, formerly HBM). The objective of this evaluation was to assess the ability of the system to continuously monitor and record strain and acceleration responses in a structure under mechanical loads. Unlike event-triggered impact detection, this experiment focused on continuous acquisition of strain gauge and accelerometer data, enabling real-time observation of structural deformations and dynamic responses.

**Figure 22:**
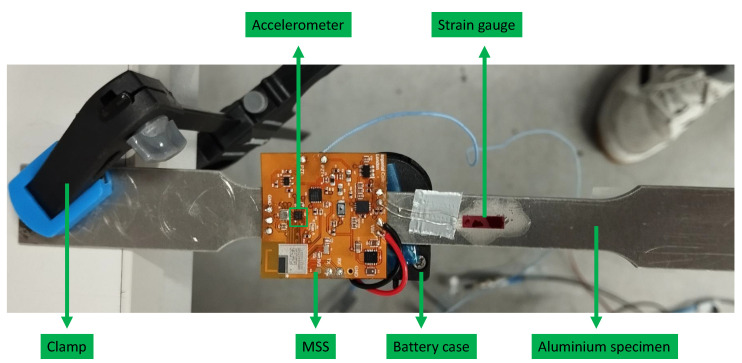
Experimental setup to compare acceleration and strain gauge measurements between the proposed MSS and a DAQ system; figure shows the top view.

To validate the system performance, an MSS was integrated with an aluminium specimen for strain and acceleration measurements. Aluminium was selected because of its relatively lower stiffness compared to composite materials, allowing for larger deformations and a wider strain range. The specimen was clamped on one side, as shown in Figure 22, and an external force was applied at the other end to induce strain and acceleration variations.

**Figure 23:**
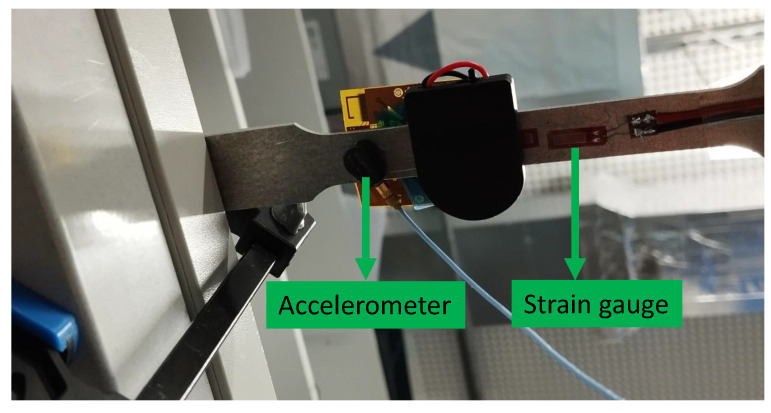
Experimental setup to compare acceleration and strain gauge measurements between the proposed MSS and a DAQ system; figures shows the bottom view.

As shown in Figure 22, a strain gauge sensor was attached to the specimen and connected to the MSS. In addition, the MSS was equipped with an on-board accelerometer (BMA400 from Bosch Sensortec), as introduced earlier. To compare the results, another strain gauge sensor and accelerometer (Model 352A24 from PCB Piezotronics) were attached to the bottom, as shown in Figure 23, and connected to the commercial DAQ system.

Subsequently, external alternating forces were applied to the specimen, and measurements were recorded from the strain gauge and accelerometer sensors using both the MSS and the commercial DAQ system simultaneously.

The next sections present the experimental results, discussing the accuracy and reliability of the strain and acceleration measurements captured by the MSS.

#### 6.4.1. Comparison of Strain Gauge Measurements

**Figure 24:**
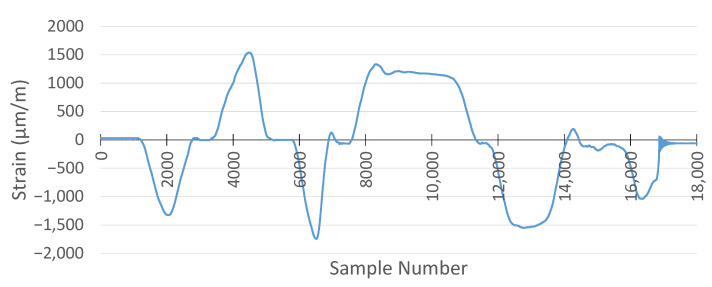
Strain gauge measurement with HBK DAQ system.

For strain gauge measurements, the sampling frequency was set to 1 kHz for both the MSS and the commercial DAQ system. The result of the commercial DAQ system is presented in Figure 24.

**Figure 25:**
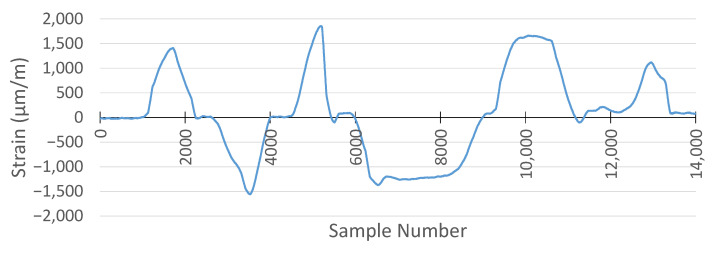
Strain gauge measurement with the proposed MSS system.

For the same applied forces, the strain measurement with the MSS is given in Figure 25. Due to the positions of the strain gauge sensors, one of them was attached on the top side, while another was attached on the bottom side of the specimen, and the strain measurement obtained using the MSS was inverse relative to those recorded by the commercial DAQ, as expected. This is because one strain gauge sensor measured tensile strain, while another measured compressive strain under the same load, and vice versa. Therefore, when the strain measurement of the MSS was reversed, the pattern and peak values of the measurement were almost the same as those of the commercial DAQ.

When comparing the results from the MSS and the commercial DAQ system, another critical point observed was that the sample numbers taken were different for the same measurement period, although both systems had the same sampling frequency of 1 kHz. While the commercial DAQ system recorded 18,000 samples, the MSS captured 14,000 samples in the experiment performed.

The reason is that the MSS records strain values until its predefined buffer becomes full, and then data are transmitted wirelessly to be processed. In this period of wireless data transmission, there is a very short delay in processing the strain values. Therefore, the MSS had a lower sample number for the same measurement period than the commercial DAQ system. However, as observed from the measurements in Figure 24 and Figure 25, the overall strain patterns and peak values are nearly identical. These short delays caused by each data packet transmission taking place wirelessly resulted in a reduction in the sampling frequency for the MSS.

**Figure 26:**
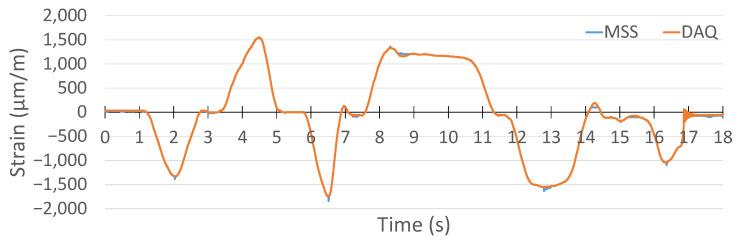
Comparison of strain gauge measurement between the proposed MSS and HBK DAQ system.

To compare the results, the measurement obtained by the MSS was reversed and stretched in the same time domain as with the commercial DAQ system. The comparison is shown in Figure 26. The two signals exhibit closely matching waveforms with similar amplitudes and peak strain values.

#### 6.4.2. Comparison of Acceleration Measurements

For comparison of the acceleration results between the MSS and the commercial DAQ systems, external forces were applied to vibrate the specimens to observe their decay patterns. The comparison was achieved in the Z axis, because model 352A24 from PCB Piezotronics measures only the acceleration in the Z axis, while the on-board accelerometer in the MSS can measure the acceleration on three axes (X, Y, and Z).

Furthermore, the sampling frequency of the commercial DAQ system to measure the acceleration was 1 kHz, while the output data rate of the on-board accelerometer was set to 800 Hz, which is the allowable maximum rate.

**Figure 27:**
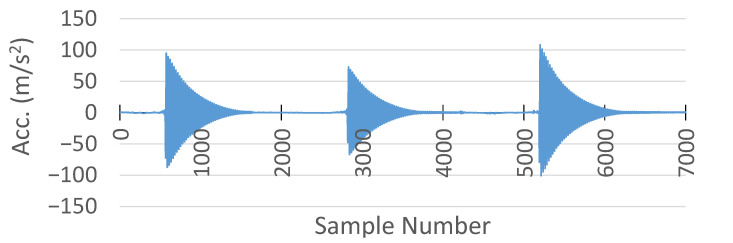
Acceleration measurement with HBK DAQ system.

The acceleration measurement collected from the commercial DAQ system is shown in Figure 27.

**Figure 28:**
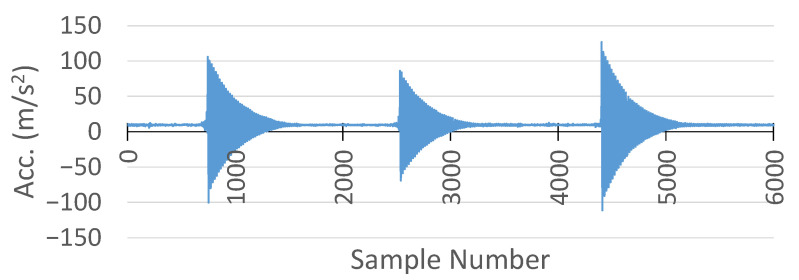
Acceleration measurement with the proposed MSS system.

Meanwhile, the acceleration recorded by the MSS in the Z axis is plotted in Figure 28. Similarly to the comparison of the strain measurements, the MSS had a lower sample number than the commercial DAQ system. The first reason was the short delay caused by the wireless communication, as discussed earlier in the strain measurement comparison. Secondly, the output data rate of the on-bard accelerometer in the MSS was already slower than that of the commercial DAQ system.

Another critical point is that there was an offset in the acceleration measurement with the MSS, as shown in Figure 28. This offset was +1 g on the Z axis, because the sensor was experiencing Earth’s gravitational acceleration [36].

**Figure 29:**
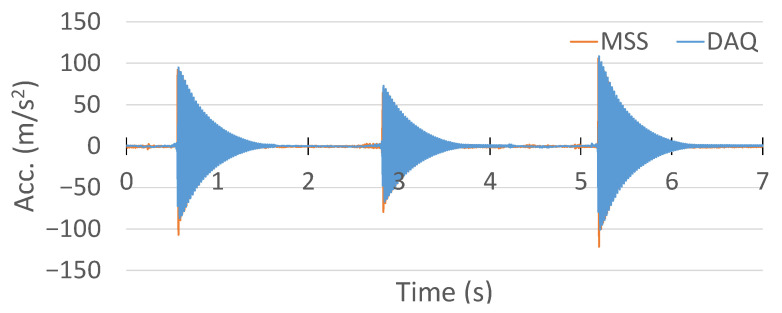
Comparison of acceleration measurement between the proposed MSS and HBK DAQ system.

**Figure 30:**
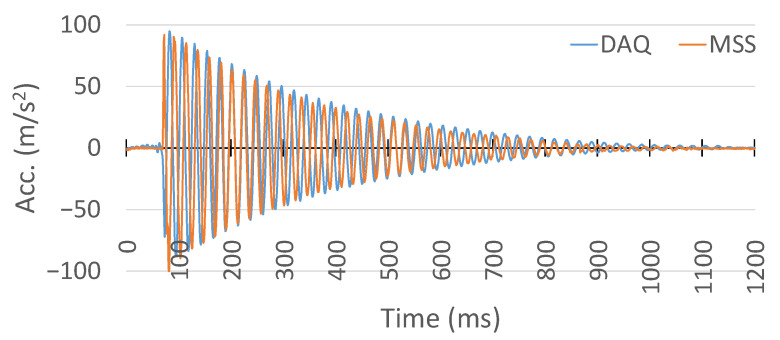
A close-up comparison of acceleration measurement between the proposed MSS and HBK DAQ system.

Therefore, to compare the results, the offset in the MSS acceleration measurement was removed and stretched in the same time domain with the commercial DAQ system. Their comparison in the same time domain is shown in Figure 29. In addition, a close-up comparison is also provided in Figure 30.

Although the MSS exhibited higher amplitudes in the initial peaks in the negative direction, both the MSS and commercial DAQ signals shared a similar oscillatory profile and decay pattern, validating the MSS acceleration output. This difference in peak amplitudes could be attributed to variations in the accelerometer mounting locations and substrate properties, which may influence the signal response.

In summary, the proposed MSS showed reliable and accurate performance in capturing both strain and acceleration measurements compared to the commercial DAQ system. Minor variations in sampling frequency were observed because of short wireless transmission delays. The MSS consistently reproduced the essential waveforms, decay patterns, and peak values measured by the commercial DAQ system. In general, the MSS demonstrates reliable real-time DAQ and serves as a suitable and cost-effective option for continuous SHM.

### 6.5. Measurement of Current Draw

The achievement of high energy efficiency is critical to extending the operational lifetime of the proposed sensing system. To quantify the power demands of the MSS under different usage scenarios, current consumption was measured using a Keysight 34465A digital multi-meter in three different operational modes that are representative of typical node behavior, as detailed below:

1. **Sleep Mode:** The sensor node persists in a low-power condition, with the wireless communication modules inactive and the sensing functions suspended. This mode represents the baseline energy usage during idle periods without external events.
2. **Time Synchronization:** During network initialization or periodic resynchronization, the MSS engages in wireless communication to align with the shared clock. The communication and synchronization processes result in moderate power consumption relative to the sleep mode.
3. **Active Mode:** When an external impact is detected, the sensor node enters a fully active mode, acquiring timestamp information, sampling all sensor data, and transmitting them wirelessly. Although this mode draws the highest current, it is sustained only briefly—about a few milliseconds.

Table 1 presents the measured current consumption for each of these operating states, offering insight into the power profile of the MSS and highlighting its suitability for low-power, long-term deployment scenarios.

### 6.6. Wireless Communication Range Evaluation

Reliable data transmission within a wireless sensor network requires a sufficient communication range between the peripheral nodes and the central node. The effective range depends on several parameters, including the selected modulation scheme (e.g., standard 1M PHY or long-range coded PHY), transmission power, and environmental propagation characteristics. Factors such as signal attenuation, reflection, and absorption, especially common in obstructed or indoor environments, can significantly degrade signal strength. In addition, antenna characteristics and orientation play a critical role in determining the achievable range [40,41].

Taking these parameters into account, the communication performance of the proposed system was assessed experimentally. All range tests were conducted in an open outdoor environment with direct line-of-sight paths between the nodes to minimize interference from multi-path propagation or structural obstacles.

To determine the maximum communication distance, a stable wireless link was first established between the central and peripheral nodes. The nodes were then gradually moved apart while the connection status was continuously monitored. The test was terminated when the connection failed to re-establish, as indicated by an on-board LED on the MSS node.

This range assessment was carried out under different configurations, including two transmission power levels (0 dBm and +8 dBm) and three PHY modulation schemes: coded PHY, 1M PHY, and 2M PHY. The lower transmission power served as a baseline to evaluate receiver sensitivity, while the increased power level helped quantify the range extension achievable under higher output conditions. Comparison across the PHY schemes provided further information on the trade-offs between the data rate and communication distance.

Table 2 presents the measured communication ranges for each combination of transmission power and modulation scheme.

## 7. Conclusions

This work presented a miniaturized, WSN-based multi-sensor system intended for on-board SHM of aerospace structures. The primary goal was to produce a sensing platform that includes various sensor types, low power consumption, and wireless connectivity. The developed system features a compact and flexible PCB measuring 3 cm × 3 cm and weighing approximately 0.5 g. It integrates PZT, strain gauge, accelerometer, and environmental sensor devices for comprehensive structural monitoring. This arrangement allows the system to capture both mechanical and environmental data that influence structural integrity. The hardware design ensures minimal weight and compact dimensions, thus addressing aerospace requirements where additional mass or size might degrade performance or limit the number of feasible sensing nodes.

The use of a single-RF microcontroller featuring Bluetooth LE enables the creation of a low-power, spatially distributed sensor network that reduces wiring needs and simplifies sensor placement. Through a star topology design, several sensor modules communicate with a central node, which then relays data to a base station for recording and analysis.

A key element of the proposed setup is the customized GUI designed for data processing and visualization. This interface simplifies interaction with the system, promoting user-friendliness and enabling efficient data analysis. In addition, different GUI configurations were customized to accommodate various experiments, thus offering versatility in DAQ and processing.

Experimental evaluations demonstrated the reliability of the developed platform. First, four MSSs were placed at various positions on a composite panel to measure PZT, acceleration, temperature, pressure, and humidity data. The system responded promptly to impact events and then transmitted all acquired data captured from the sensors to the central device.

Second, the MSS demonstrated its versatility in measuring real-time strain and acceleration by applying it to a strain gauge mounted on an aluminium specimen. Calibration procedures effectively eliminated offsets from the Wheatstone bridge, ensuring accurate strain measurements. Comparative tests with a commercial DAQ system showed that the MSS captured structural responses with closely matching signal patterns and peak values. Minor discrepancies in sample count, due to wireless transmission delays, had no significant impact on the accuracy of the measurement. These results confirm that the MSS is capable of reliable continuous monitoring.

The current consumption was evaluated by measuring current draw across various operational modes, including sleep mode, time synchronization, and active mode. The results confirm that the system is well suited for long-term SHM applications with minimal power requirements. In addition, range testing was performed to examine the system’s Bluetooth LE communication under various transmission power rates (0 and +8 dBm) and modulation schemes (CODED, 1M, and 2M PHY). The findings confirm that the MSS provides reliable wireless coverage suitable for aircraft and structural applications.

These results confirm that the proposed multi-sensor platform has already progressed beyond the conceptual and laboratory model stage and is fully prototyped on a miniaturized, flexible PCB. The current version of the system has undergone functional validation through a series of laboratory experiments, demonstrating its capability in real-time multi-sensor data acquisition, wireless communication, and accurate signal processing. In the next step, the developed MSS is ready to be tested in real-world applications, including on-board SHM for aircraft.

The miniaturized flexible PCB described in this work is particularly promising for ‘smart skin’ implementations. Using a flexible substrate and compact sensor integration, the platform is designed to accommodate complex or curved surfaces on aerostructures. This feature supports distributed sensing across large areas, providing detailed measurements of vibration, strain, and environmental variables.

Another beneficial extension is to incorporate energy harvesting modules into the platform, such as vibration-based or solar-based solutions. These modules could supplement or even replace conventional batteries, improving system longevity and reducing maintenance demands for large-scale sensor deployments. In combination with the flexible sensor board, such energy-harvesting approaches make it possible to create self-sustaining ‘smart skin’ systems capable of continuous SHM.

## Figures and Tables

**Table 1:** Characterized current draw in different operational states.

Operational Mode	Current Consumption
Sleep Mode	55 μA
Time Synchronization	1.3 mA
Active Mode	7.5 mA

**Table 2:** Achieved communication range under various TX Power levels and modulation schemes.

TX Power	Coded PHY	1M PHY	2M PHY
0 dBm	60 m	28 m	15 m
+8 dBm	160 m	75 m	40 m

## Data Availability

The data will be made available upon reasonable request.
